# Eco-Challenges of Bio-Based Polymer Composites

**DOI:** 10.3390/ma2030911

**Published:** 2009-08-10

**Authors:** Maurizio Avella, Aleksandra Buzarovska, Maria Emanuela Errico, Gennaro Gentile, Anita Grozdanov

**Affiliations:** 1 Institute for Chemistry and Technology of Polymers, ICTP-CNR, 80078 Pozzuoli, Naples, Italy; E-Mails: bors@ictp.cnr.it (M.E.E.); gengenti@ictp.cnr.it (G.G.); 2 University Ss Cyril and Methodius, Faculty of Technology and Metallurgy, Rugjer Boskovic 16, 1000 Skopje, Macedonia; E-Mails: abuzar@tmf.ukim.edu.mk (A.B.); anita@tmf.ukim.edu.mk (A.G.)

**Keywords:** biopolymer, poly(lactic acid), natural fibers, nanocomposites

## Abstract

In recent years bio-based polymer composites have been the subject of many scientific and research projects, as well as many commercial programs. Growing global environmental and social concern, the high rate of depletion of petroleum resources and new environmental regulations have forced the search for new composites and green materials, compatible with the environment. The aim of this article is to present a brief review of the most suitable and commonly used biodegradable polymer matrices and NF reinforcements in eco-composites and nanocomposites, with special focus on PLA based materials.

## 1. Introduction

In the last decades biopolymers prepared from renewable resources have attracted great attention in both the academic and industrial worlds. In fact, renewable sources of polymeric materials reinforced with lignocellulosic fibers derived from annually renewable resources, offer an answer to maintaining sustainable development of economically and ecologically attractive materials with respect to ultimate disposability and raw materials use. In recent years, scientists and engineers have been working together to use the inherent strength and performance of the fibers and nanoparticles in combination with natural green polymers to produce a new class of bio-based composites. The special challenges for this type of bio-composites are their eco attributes, that make them environmentally friendly, completely degradable and sustainable.

Poly(lactic acid) (PLA) is a class of crystalline biodegradable thermoplastic polymer with relatively high melting point and excellent mechanical properties. Recently PLA has been highlighted because of its availability from renewable resources such as corn and sugar beets. PLA is synthesized by the condensation polymerization of d- or l-lactic acid or ring-opening polymerization of the corresponding lactide [1,2].

Under specific environmental conditions, pure PLA can degrade to carbon dioxide, water and methane over a period of several months to two years, a distinct advantage compared to other petroleum plastics that need much longer periods. Advanced industrial polymerization technologies have been developed to obtain high molecular weight pure PLA, which leads to a potential for structural materials with enough lifetime to maintain mechanical properties without rapid hydrolysis.

The final properties of PLA strictly depend on its molecular weight and crystallinity. PLA has been extensively studied as a biomaterial in medicine, but only recently it has been used as a polymer matrix in composites. In fact, PLA resins are nowadays marketed for different applications. In 2002, Cargill-Dow LLC started up a commercial PLA plant, with the aim of produce PLA fibers for textiles and nonwovens, as well as PLA films for packaging applications and rigid containers.

The aim of this review is to examine the state of the art regarding PLA-based materials and in particular, eco-composites realized with natural fibres, agricultural wastes, micro and nanocomposites in order to define the real final possibilities for these materials to be accepted on the market.

## 2. Results and Discussion

### 2.1. Fiber-reinforced PLA composites

Interest in biodegradable polymers and natural fiber-reinforced polymers has recently grown because of increasing environmental concerns. Natural fiber reinforcements could considerably lower the price of bio-based composites that is still the higher barrier for their wider application. Moreover, since they derive from renewable sources, they can represent environmental friendly alternatives to conventional reinforcing fibers (glass, carbon, Kevlar^®^). Further advantages of natural over synthetic fibers are good specific mechanical properties, reduced tool wear, enhanced energy recovery, biodegradability, etc. Besides this, the natural fibers can also affect the mechanical properties of bio-matrices.

It has been reported that tensile and flexural modulus results could be improved by increasing the content of cellulose or cellulose based reinforcements in PLA based composites, whereas tensile and flexural strength remain practically unchanged or were worsened [3,4,5]. With regards to impact properties, the toughness results were impaired for PLA composites reinforced with cellulose [4], whereas small improvements are recorded with the addition of cotton or kenaf fibers [6,7].

These results can be justified considering that tensile and flexural modulus of a composite are strongly dependent on the modulus of the components but only slightly sensitive to the interfacial adhesion. In fact, modulus is measured at very small strain when simple physical contact of components is sufficient to transfer the stress. As a matter of fact, the inclusion of a rigid phase, such as cellulose fibers, is able to increase the polymer stiffness.

Conversely to the modulus, tensile and flexural strength are very sensitive to the fibre/matrix interfacial adhesion. In fact, these parameters refer to nont-negligible deformations, so that the interface plays a crucial role in transferring the stress from the matrix to the fibrous phase.

In fact, it is well known that the enhancement of performance in multicomponent polymer based materials is often ascribed to a strong interfacial adhesion generated through interactions between different phases. Different surface properties between fibers (highly polar) and common polymer matrices (non-polar and hydrophobic) require the set up of a proper strategy to improve the fiber/polymer compatibility and their interfacial adhesion. Without such a strategy, unstable NF/polymer interfaces are generated; likewise the poor ability of the polymer to completely wet the fibers prevents their homogeneous dispersion. In this case the overall properties of the composite can be unsatisfactory.

In particular, regarding the mechanical response of materials, as an example, when an external load is applied to composites, the load is transferred to the fibers nearest the surface and continues from fiber to fiber via matrix and interface. Then, a weak interface induces an ineffective load distribution and the potential reinforcement effect of fibers remains underexploited. On the contrary, a strong interface can assure an efficient transfer of the applied load to fibrous reinforcements through the matrix with a consequent improvement of composite mechanical behavior.

Finally, the toughness mechanism in fiber reinforced composites can be qualitatively described by considering that the energy dissipated by the fracture can be evaluated as the addition of three main components: matrix fracture energy, fiber fracture energy and an interaction term accounting for debonding and pull-out phenomena. The fiber fracture energy and the interaction term are negligible for short or weak fibers; in this case the overall toughness of the composite is worsened independently from the interfacial adhesion. On the contrary, both these terms can exceed the matrix contribution by adding long and/or strong fibers, thus justifying an improvement of the toughness as a function of the interfacial strength.

As a matter of fact, the promotion and the optimization of a strong fibers/polymer interfacial adhesion play a critical role and represent the focus of much research regarding the realization of composites. Nowadays two approaches are considered effective for this purpose: the surface modification of fibers and the use of an appropriate compatibilizing agent.

In the last years different natural fibers have been employed in order to modify the properties of PLA. Up to now, the most studied natural fiber reinforcements for PLA were kenaf [6,7,8], flax [9,10], hemp [11], bamboo [12], jute [13] and wood fibers [14]. Besides conventional natural fibers, recently reed fibers have been tested in appropriate PLA composites in order to improve the tensile modulus and strength [14].

Generally, the mechanical properties of natural fiber reinforced composites were improved by using surface modified fibers. Huda *et al.* worked on kenaf fiber reinforced PLA laminated composites prepared by compression molding using the film-stacking method [6]. Their goal was to evaluate the mechanical and thermal properties of these composites as a function of modification of the kenaf fiber using alkalization and silane-treatments. They found that both silane-treated fiber reinforced composite and alkali treated fiber reinforced composite offer superior mechanical properties, compared to untreated fiber reinforced composite. The alkali- followed by silane-treated fiber reinforced composite also significantly improved mechanical properties. Moreover, morphological studies by scanning electron microscopy demonstrated that better adhesion between the fiber and the matrix was achieved. It was found that standard PLA resins are suitable for the manufacture of kenaf fiber reinforced laminated biocomposites with useful engineering properties.

The mechanical properties of PLA composites reinforced with Cordenka^®^ rayon fibers and flax fibers which are examples for completely biodegradable composites were tested and compared. The samples were produced using injection moulding. The highest impact strength of 72 kJ·m^-2^ and tensile strength of 58 MPa were found for Cordenka^®^ reinforced PLA at 30 wt% fiber-mass content. The highest Young's modulus of 6.31 GPa was found for the composite made of PLA and flax. A poor adhesion between the matrix and the fibers was shown for both composites using SEM. The promising impact properties of the presented PLA/Cordenka^®^ composites show their potential as an alternative to traditional composites [9].

The effects of the alkali treated natural fibers on the mechanical properties of PLA/hemp fibers were studied by Hu and Lim [11]. They fabricated completely biodegradable composites of PLA reinforced with short hemp fibers by using the hot-press method. The results show that the composite with 40% volume fraction of alkali treated fiber possessed the best mechanical properties. The tensile strength, elastic modulus, and flexural strength of the composite with 40% treated fiber were 54.6 MPa, 8.5 GPa, and 112.7 MPa respectively, which are much higher than those of neat PLA. The composites have lower densities, which were measured to be from 1.19 g·cm^-3^ to 1.25 g·cm^-3^. Surface treatment of the natural fibers was used also to improve the impact resistance of the PLA-based composites. Bamboo fiber (BF) reinforced PLA composites were prepared in order to improve the impact strength and heat resistance of PLA [12]. Three different types of BF were extracted from raw bamboo by either sodium hydroxide treatment or steam explosion in conjunction with mechanical processing. They were designated as “short fiber bundle,” “alkali-treated filament” and “steam-exploded filament,” respectively. Composite samples were fabricated by injection molding using PLA/BF pellets prepared by a twin-screw extruding machine. Among them, the highest bending strength was obtained when steam-exploded filaments were put into PLA matrix. Impact strength of PLA was not greatly improved by addition of short fiber bundles as well as both filaments. In order to improve the impact strength of PLA/BF composites, PLA composite samples were alternatively fabricated by hot pressing using medium length bamboo fiber bundles (MFB) to avoid the decrease in fiber length at fabrication. Impact strength of PLA/MFB composite significantly increased, in which long fiber bundles were pulled out from the matrix. The addition of BF improves thermal properties and heat resistance of PLA/BF composites due to the constraint of deformation of PLA in conjunction with crystallinity promoted by annealing treatment (T=110 °C for 5 h).

Recently an innovative method used to improve the mechanical properties of PLA based composites is utilization of continuous hybrid fiber reinforced composite yarn obtained by a micro-braiding technique [13]. Completely naturally-derived micro-braided-yarn was fabricated by using thermoplastic biodegradable PLA resin fiber as the resin fiber and jute spun yarn as the reinforcement. Using jute spun yarn/PLA micro-braided-yarn, continuous natural fiber reinforced biodegradable resin composite plates was molded by hot press molding with various molding conditions.

The suitability of wood fibers (WF) as natural reinforcements in PLA based composites has been also demonstrated in comparison with WF reinforced polypropylene composites [14]. Results showed that 40 wt% of WF are able to induce an improvement of about 200% of the flexural modulus of PLA, comparable to the improvement obtained for polypropylene composites reinforced with the same amount of WF. Flexural strength of WF/PLA composites is also enhanced, going from about 100 MPa for the neat resin to about 115 MPa for PLA based composites reinforced wit 20-40 wt% of WF.

The effects of surface treatment of pineapple leaf fibers (PALF) on the performance of the natural fiber-reinforced renewable composites was studied for laminated composites prepared by compression molding using the film stacking method [15]. The results have shown that mechanical properties of the PLA laminated composites were remarkably improved after chemical treatment. It was found that both silane- and alkali-treated fiber reinforced composites offered superior mechanical properties compared to untreated fiber reinforced composites. Differential scanning calorimetry (DSC) results suggested that surface treatment of PALF affects the crystallization properties of the PLA matrix.

A new biocomposite based on chicken feather fibers (CFF) and PLA has been fabricated for the first time by the melting compound method. The tensile modulus and elongation at break results of the PLA samples was improved by adding small amounts of CFF. This behavior is ascribed to the good adhesion and interactions between the CFF and PLA matrix [16]. Also, silkworm silk fibers are recognized as reinforcements in PLA natural fiber composites for tissue engineering application. Due to the fact that the silk fiber surface bonds well with the polymer matrix, these fibers can be good candidate, as reinforcements for the development of polymeric scaffolds for tissue engineering applications [17].

Sometimes, in order to provide a good adhesion between biodegradable matrix and natural fiber reinforcements, the use of adhesion promoter is necessary. For example in PLA/cotton fiber reinforced composites the use of lignin as adhesion promoter, resulted in improved tensile strength and modulus (improvements of about 10% and 25%, respectively) but decreased impact properties [5].

**Figure 1:**
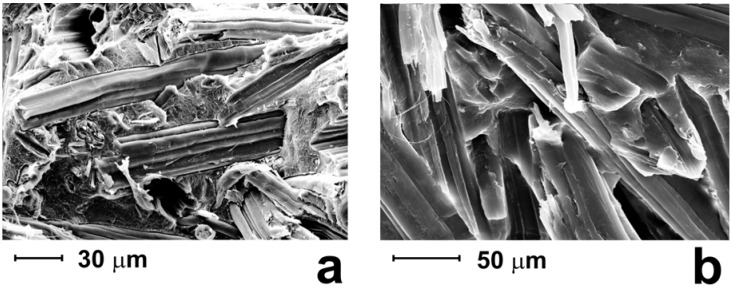
SEM micrographs of PLA/kenaf composites containing 30 wt% of kenaf fibres: (a) uncompatibilized materials; (b) compatibilized materials.

A more efficient way to improve the adhesion between fibers and biodegradable resin could be provided by the utilization of coupling agents. In PLA/kenaf fiber composites a suitable reactive coupling agent was obtained by grafting maleic anhydride onto PLA [7,8]. PLA-based composites were prepared by a proper *in situ* reactive compatibilization. Namely, low amount of PLA grafted with maleic anhydride (PLA-g-MA) was added to the composite components. Maleic anhydride groups grafted onto PLA chain are reactive with respect to hydroxyl groups present on the fiber surface. In this way, interactions between hydroxyl and maleic anhydride groups are responsible for *in situ* formed grafted species that are able to effectively compatibilize PLA/fiber composites. In fact, morphological analysis carried out on these systems revealed that a significant enhancement of the adhesion level between the fibers and the matrix is observed for PLA/kenaf composites compatibilized by using 5 wt% PLA-g-MA. In Figure 1 SEM micrographs of uncompatibilized and compatibilized PLA/kenaf composites containing 30 wt% of kenaf fibers are shown.

**Figure 2:**
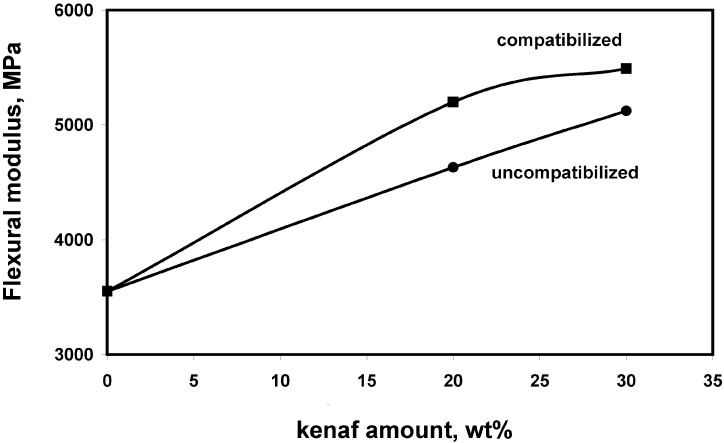
Flexural modulus of PLA based composites as a function of the kenaf content and the compatibilization.

**Figure 3:**
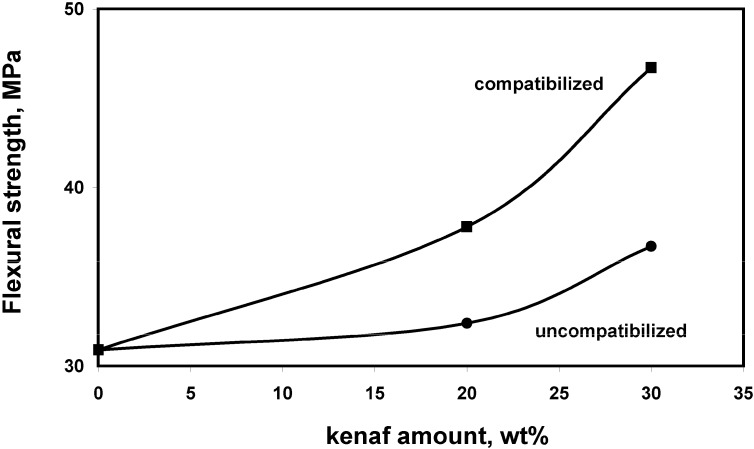
Flexural strength of PLA based composites as a function of the kenaf content and the compatibilization.

**Figure 4:**
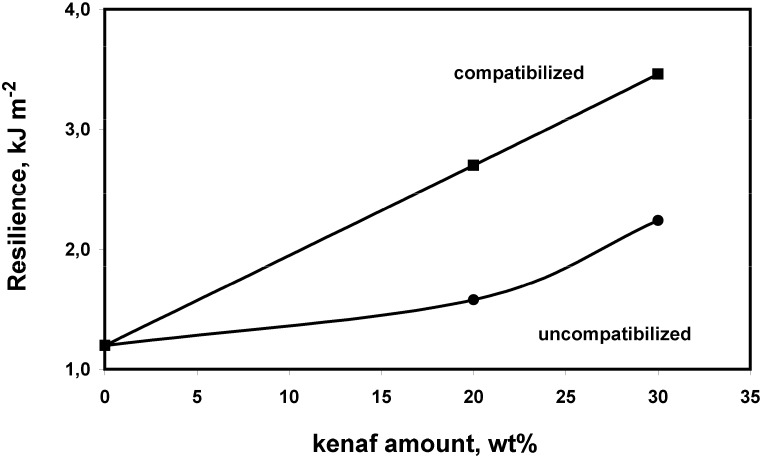
Resilience of PLA based composites as a function of the kenaf content and the compatibilization.

Mechanical properties of neat PLA, uncompatibilized and compatibilized PLA/kenaf composites are shown in Figure 2, Figure 3 and Figure 4. Significant improvements of the flexural modulus, up to 55%, were recorded as a function of kenaf fiber content. Higher modulus values were obtained in presence of the reactive coupling agent. The same behavior has been observed for the flexural strength, the highest values being obtained for compatibilized composites reinforced with 30 wt% of kenaf fibers. Also the resilience of neat PLA was improved, up to about 190%, for compatibilized composites.

### 2.2. PLA-Agricultural waste composites

Renewable agricultural waste lignocellulosic materials and bio-based polymer matrices provide an attractive eco-friendly quality as well as the environmental sustainability to the resulting natural fiber reinforced bio-based composites. Recently, the interest in agricultural wastes as a substitute for wood-based raw materials has increased significantly.

Water bamboo husk is one of major agricultural wastes in Taiwan. The powder obtained from the water bamboo husk was added to PLA to form novel reinforced biodegradable composites [18]. Morphologies, mechanical properties, and heat resistance of these water bamboo powder reinforced composites were investigated. The results indicate that the char yields were increased as plant powder was incorporated to PLA. In addition, the mechanical properties were also enhanced due to the addition of powders. The increments of storage modulus of PLA were about 50%-200%. Moreover, the increments of loss modulus of PLA were about 70%-200%. On the other hand, the Tg of PLA was slightly decreased by the addition of powder, and this may improve the brittle characteristics of PLA. Furthermore, it was concluded that this type of reinforced PLA would be more environmental friendly than the artificial additive-reinforced one.

Other agricultural waste that could be considered as potential reinforcing filler in semi-crystalline thermoplastic bio-based polymer composites is also, the rice straw. Global paddy production reached 628 million tons in 2005 with an additional 1% increase in 2006. The rice straw fiber has the similar chemical composition as other natural fibers used in composites. Generally, whole rice straw is composed of rice husks, leaf sheathes, straw leaf blades, straw steam and knots, and straw roots. All fiber components have various chemical constituents, especially cellulose and residual ash content, that may contribute differently to the properties of the rice straw reinforced composites. The main carbohydrate components of rice straw are hemicellulose, cellulose and lignin. Due to the rice straw resistance to bacterial decomposition, as well as high content of silica (up to 20 wt%), the rice straw is suitable as a filler in building composites. In the framework of the ECO-PCCM project supported by the EU FP6-INCO program, eco-composites based on PLA, PHBV and PP reinforced with rice straw were designed and studied [8,19,20]. The properties of the obtained PLA/rice straw composites are comparable to commercially available polypropylene composites reinforced with 20 wt% of glass fibers. Moreover, processing can easily be carried out in one step below a critical fiber volume.

### 2.3. PLA nanocomposites

The incorporation of nanoparticles in certain biodegradable matrices could significantly affect the crystallization behavior, morphology, mechanical properties as well as biodegradation [21]. Also, water barrier and antimicrobial properties are very important characteristics when using biodegradable polymers for special purposes like packaging. For example, PLA nanocomposites based on different types of nanoclays such as Cloisite^®^ Na+, Cloisite^®^ 30B and Cloisite^®^ 20A are effective in improving the water vapor barrier properties and bacteriostatic function against certain microbes [21].

The nanocomposites based on PLA are of special interest for medical purposes. Special attention has been paid to novel nanomaterials capable of facilitating the biorecognition of an anticancer drugs. For example, blending of titanium dioxide (TiO_2_) nanoparticles and polylactide (PLA) nanofibers has been adopted as a new nanomaterial that lets drug molecules readily self-assemble on the surface of the nanocomposite. These novel nanocomposites imply some potential valuable application as a kind of drug carriers in view of the respective good biocompatibility of PLA and large surface area of the nanoparticles [22].

Tissue engineering has become an alternative method to traditional surgical treatments for the repair of bone defects, and an appropriate scaffold supporting bone formation. For these purposes special nano-sized demineralized bone powders with PLA were electrospinned for engineering bone [23]. Electrospinning is known as a novel fabrication method to form nanofibrous scaffolds for tissue-engineering application. Previously, many natural biopolymers of protein have been electrospun. In some cases the introduction of special components, like keratin or gelatin electrospinned with PLA result in nanofibers that better adhere to the cells [24].

In some cases the properties of nanocomposites depend on the intercalated or exfoliated structures. In order to improve the intercalated or exfoliated structures of the nanocomposites, twin-screw extrusion is usually employed. Effectively increase of the binding force between the phases could be improved by addition of compatibilizer. For example in poly(lactic acid)/montmorillonite (PLA/MMT) nanocomposites prepared by twin-crew extrusion, the addition of polycaprolactone (PCL), as a compatilizer, can improve the thermal properties [25].

Electrospinning is also a straightforward method for producing anti-ultraviolet polymer fibers. The new types of anti-ultraviolet nanofibers are produced by PLA and benzophenone-12, different anti-ultraviolet nanoparticles (TiO_2_) and others elements (Chemfos^®^-168) [26]. Certain nanocomposite systems, such as PLA-clay nanocomposites could be also used for developing fibers. The modified clays show good compatibility with PLA matrix, inducing a positive effect on the mechanical properties [27].

Nanotubes are nowadays commonly used in nanocomposites. In some cases these nanofillers could form a conductive network structure in appropriate polymer matrix, which is the key for liquid sensing. Liquid sensing properties have been identified in PLA/multi-walled carbon nanotube (MWNT) composites. Very interesting properties, like changing of the electrical properties upon solvent contact have been observed [28].

In order to improve the thermal and mechanical properties of the biodegradable matrix sometimes it is necessary to modify nanosized particles. In PLA/nanosized calcium carbonate (CaCO_3_) composites, calcium stearate was used as modifier to improve the adhesion between the CaCO_3_ particles and the PLA matrix. The tensile strength and modulus values of the composite could be improved greatly without a significant loss in the elongation at break when the nanosized CaCO_3_ was incorporated up to 30 wt % [29].

Multi-walled carbon nanotubes (MWCNTs) are usually functionalized to achieve their better dispersion within the polymer matrix. Both the dispersion state and the surface functionalization of MWCNTs are very important for the thermal stability of the biopolymer. Different functionalities of the MWCNTs could have different effect on the dispersion and therefore on the thermal stability of the nanocomposites [30].

MWCNTs are used in many cases to achieve high electrical conductivity at low carbon nanotube loadings. When only 0.5 parts per hundred parts of resin (phr) modified multi-walled carbon nanotubes are added to low-crystalline PLA, the surface resistance of the composite could fell by 10-13 order. The effect is usually realized due to the enhanced MWCNT dispersion by covalent or hydrogen bonding between modified multi-walled carbon nanotube and PLA. The degree of crystallinity of PLA can influence the electrical property of MWCNT/PLA composites apparently as well [31].

Water-crosslinking technique is adopted to improve the physical characteristics of the nanocomposites. The PLA composites prepared by this approach show that crystalline PLLA can be transformed to a natural semicrystalline poly(lactic acid) after a water-crosslinking reaction. The composites after water-crosslinking treatment, usually exhibit better mechanical properties than the non-crosslinked composites because of strong chemical intra-molecular bonding. Also the thermal degradation temperatures of the nanocomposite increase with the increasing of water-crosslinking time [32].

In some cases the introduction of nanoparticles in biopolymer could significantly affect the rate of hydrolytic degradation. Generally the degradation rate constants are higher for amorphous PLA and its composites than semicrystalline PLA and its composites. In addition, the proper treatment of nanofillers could have certain influence on their hydrophilicity change as well as on the degradation rate constants of the nanocomposites [33].

The preparation procedures could also affect the final properties of the nanocomposites. Frequently, solution intercalation and melt intercalation methods are common procedures for the preparation of nanocomposites. For melt intercalation procedure, usually the single–screw extruder or twin-crew extruder are used.

PLA/organo-montmorillonite (OMMT) nanocomposites were prepared by using solution intercalation and melt intercalation methods. In some cases, composites prepared by solution intercalation methods show better dispersion of the nanoparticles within the polymer matrix when compared to the melt intercalation processes. It is interesting to note that optimum flexural properties could be achieved at low nanoparticles loadings, up to 1 wt% [34].

The degree of dispersion could be additionally influenced by the modification of the nanoparticles and by the optimization of the processing conditions. Nanobiocomposites of poly(lactic acid) with 3-5 wt% organically modified montmorillonite were prepared by melt compounding using either a miniextruder or an internal mixer [35]. Results showed that varying processing conditions can significantly improve the dispersion of the nanoparticles, thus influencing rheological, optical and mechanical properties of PLA nanocomposites. Successfully embedded purified carbon nanotubes (CNTs) in a PLA matrix showed better dispersing and properties in the CNTs PLA nanocomposites [36].

*In-situ* polymerization methods are also employed to prepare nanocomposites. The nanoparticles take active role in ring opening polymerization process of l-lactide. In these processes a fine dispersion is achieved at low nanoparticle loadings. The dispersion could be improved by the surface modified nanoparticles. For example TiO_2_/PLA composites with different contents of TiO_2_ have markedly improved thermal and mechanical properties when the content of TiO_2_ is 3 wt% due to the surface modification of nanoparticles and their finer dispersion [37].

PLA/organo-montmorillonite nanocomposite prepared by this approach, under continuous microwave irradiation of the organo-montmorillonite nanoparticles show significant increase of the tensile strength and the elongation at break when compared with pure PLA. Following this, certain improvements of thermal stability could be identified.. The introduction of the OMMT makes the tensile fracture of the nanocomposite change from brittle rupture to ductile rupture [38].

Modified surfactant free emulsification method is another common approach for successful fabrication of PLA/nanocomposites. The use of proper organic solvent in emulsions influences the reduction of the particles sizes. Poly(d,l-lactide)/apatite nanocomposites were successfully fabricated through this modified surfactant-free emulsification method [39].

The presence of nanoparticles could affect the crystallization process as foreign bodies can accelerate the nucleation process of polymer resin. Quantitative analysis of isothermal crystallization kinetics of PLA/clay nanocomposite has brevelead that the crystallization rate constants are usually higher in PLA nanocomposites. Various nanoparticles could be found as nucleating agents for PLA. Recently, the use of novel biobased carbon nanospheres was found to accelerate the rate of crystallization of PLA [40].

Recently, also novel nanocomposites have been recognized. Poly(lactic acid) and nanodiamond (ND) powder composites for potential bio-engineered applications have been fabricated for the first time by using melting compound methods. Due to the unique ND bridge structures and homogenous dispersion, as well as good interfacial bonding with PLA, the mechanical properties of PLA could be considerably improved by incorporating ND powder [41].

### 2.4. PLA-Composites with microparticles

Various fillers with non-nanoscale particle dimensions could also be used to modify the properties of PLA. A common approach to incorporate them into the polymer matrix is the already mentioned ring-opening polymerization of lactide.

In some cases, lactic acid could be graft-polymerized onto the filler surface. A series of hydroxyapatite (HA)/PLA composites were prepared using this kind of filler particles modification. This approach provides good dispersion of filler particles in PLA matrix [42].

Large amounts of stable β-anhydrite II (AII), a specific type of dehydrated gypsum and a by-product of lactic acid production process, can be melt-blended with bio-sourced and biodegradable PLA to produce economically interesting novel composites with high tensile strength and thermal stability. Due to PLA’s sensitivity towards hydrolysis, the utilization of β-anhydrite II (All) as filler is a prerequisite. Good filler dispersion throughout the polyester matrix, make these composites interesting in biodegradable rigid packaging or technical applications [43]. Different modifiers can be also used to increase the toughness of these composites. For example the addition of impact modifiers based on ethylene copolymer leads to remarkable thermo-mechanical performances of these composites.

The toughness of these systems is also modified by addition of selected low molecular weight plasticizers [bis(2-ethylhexyl) adipate and glyceryl triacetate] and polymeric adipates with different molecular weights. The incorporation of plasticizers provides easier processing as well as better performances of the composites [44]. Similar plasticizers like acetyl tributyl citrate (ATBC) and poly(1,3-butylene adipate) (PBA) are used in preparation of PLA/carbon black (CB) high performance composites [45].

Some fillers containing specific functional groups are a good base for preparation of composite membranes. For example, a PLA/siloxane/calcium carbonate composite membrane containing mercapto-groups (PSC-SH) exhibits antibacterial ability. Mercapto-groups were reported to adsorb silver ions, which are well known to show antibacterial activity [46].

In the osteological field, the study of repairing and recovering processes for bone tissues has been one of the major clinical research subjects. A series of microsphere composites made from PLA and β-calcium phosphate (β-TCP), an osteogenesis material, were prepared and their potential applications in participation to the growth of new bone tissues on bone defects were estimated [47].

## 3. Conclusions

PLA-based materials are a new class of materials that in recent years has aroused an ever growing interest due to the continuously increasing environmental awareness throughout the world. They can be considered as the “green” evolution of the more traditional ecocomposites, essentially consisting of synthetic polymers based composites reinforced with natural fibres or other micro or nanofiller.

From a technological point of view, some doubt about the performance of these new materials, as well as the higher costs of biodegradable polymer matrices with respect to other polymers, still prevent their wider commercial diffusion.

With regards to the still higher commercial prices of biodegradable polymers with respect to commodities, other factors, such as the lower costs for disposal, should also be taken into account. Moreover, the price of biodegradable polymers, and in particular those derived from natural sources, is expected to further decrease in the coming years due to innovative manufacturing practices, so biodegradable composites can be considered a valid alternative to traditional composites and ecocomposites: in fact, because of their compostability, they can represent an effective solution to the waste disposal problem of polymer based materials. Their unique properties should be a solid base to develop new applications and opportunities for biocomposites in the 21st century “green” materials world.
